# Supplementary material for: Preferences for public engagement in decision-making regarding four COVID-19 non-pharmaceutical interventions in the Netherlands: A survey study
Source: PLoS One. 2023 Oct 5;18(10):e0292119. doi: 10.1371/journal.pone.0292119 (PMC10553365; doi:10.1371/journal.pone.0292119)
Supplement: S1 File — (DOCX) [file pone.0292119.s001.docx]

## Supplementary file 1 – information about the four non-pharmaceutical interventions (NPIs)

A = Nightly curfew (NC).

On 23 January 2021 a nightly curfew was implemented in the Netherlands. The outbreak situation at this point was concerning and new measures needed to be implemented to prevent a new wave of infections. This NPI implied that no one should be on the street after 9PM. Exceptions were made for e.g. work and school. There was much backlash regarding this measure within society, and scientific knowledge regarding the effectiveness of such a nightly curfew was scarce. On 28 April 2021, the Nightly curfew was abolished (1). in the Netherlands, a cohort study regularly measured public support regarding several NPIs by means of surveys. Likewise, public support for NC was measured, and results revealed that at the start of the nightly curfew, 69% of respondents who filled in the survey supported the NPI. At abomination, the percentage of respondents that supported the NC decreased to 62% (2).

B = Closure of elementary schools and daycares (CED).

Between 16 March and 6 April 2020, during the first wave of the COVID-19 epidemic in the Netherlands, elementary schools and daycares were closed as a part of the intelligent lockdown during that time. There was not much scientific knowledge available regarding the impact of such a closure on the epidemic. However, this measure was partly taken to adhere to wishes outed by citizens. Members of the public expressed concerns about the safety of employers and children when keeping schools open. Hereafter, the government decided to close the schools and daycares (1).

C = Digital Covid Certificate for events (DCC).

At the start of July 2021, during a very short period, a Digital Covid Certificate for events (e.g. sport events or theater performances) was implemented. This certificate consisted of a negative test, a vaccination certificate or a recovery-from-covid certificate. Before entering the event, visitors had to show their certificate. Due to the use of this certificate, social distancing was no longer necessary at these events, and more guests were allowed. It was more or less clear that such a certificate would decrease transmission, however, there was much public debate regarding this NPI specially about the division the certificate created in society based on vaccination status (3). Public support for the Digital Covid Certificate was measured between 26^th^ of October and 1^st^ of November in the abovementioned cohort study in the Netherlands. Between 70% and 95% of the respondents supported using DCC at concerts or festivals (2).

D = 1.5meter social distancing (1.5M).

Since March 2020 a number of basic measures were implemented. One of these basic measures was the 1.5 meter social distancing. Every citizen was strongly advised to keep this distance to others, and in some periods this measure was even mandatory, risking a fine if not adhered to. Social distancing is an evidence-based measure to reduce transmission, and was generally well accepted by citizens (4). Between 26^th^ of October and 1^st^ of November, 73% of respondents of the cohort study supported this NPI (2).

## References

1. Mouter N, de Vries M, Chorus C, ten Broeke A, Heyning N. Welke coronamaatregelen vinden Nederlanders wel en niet wenselijk bij verschillende ontwikkelingen van het virus? 2021. Available from: https://populytics.nl/wp-content/uploads/2022/06/Rapport-Coronabeleid-Samenvatting.pdf

2. RIVM. Analyses eerdere metingen: Naleving van en draagvlak voor de basis gedragsregels. 2023 Available from: https://www.rivm.nl/gedragsonderzoek/naleving-van-en-draagvlak-voor-basis-gedragsregels/analyses-eerdere-metingen

2. TU Delft. Effectiviteit van verschillende toepassingen van het Coronatoegangsbewijs. 2022. Available from: https://open.overheid.nl/repository/ronl-aa2988ce-324f-4c1c-ad39-459358e32bfe/1/pdf/effectiviteit-coronatoegangsbewijs-eindversie-tu-delft4.pdf

3. European Centre for Disease Prevention and Control. Guidelines for non-pharmaceutical interventions to reduce the impact of COVID-19 in the EU/EEA and the UK. 2020. Available from: https://www.ecdc.europa.eu/sites/default/files/documents/covid-19-guidelines-non-pharmaceutical-interventions-september-2020.pdf
