# Supplementary material for: Preferences for public engagement in decision-making regarding four COVID-19 non-pharmaceutical interventions in the Netherlands: A survey study
Source: PLoS One. 2023 Oct 5;18(10):e0292119. doi: 10.1371/journal.pone.0292119 (PMC10553365; doi:10.1371/journal.pone.0292119)
Supplement: S2 File — (DOCX) [file pone.0292119.s002.docx]

## Supplementary file 2 – Rationales behind the setup of the survey

To develop this survey, we have used several frameworks and the findings from an earlier study in which focus groups were executed regarding the same topic (1).

**Theme 1 – Reasons (not) to engage**

For Theme 1, the reasons to engage and not to engage were based on general literature on public engagement in healthcare decision-making, and the results from a former study (1). First in general, three rationales, which are described in the Introduction of our main text, describe the potential of public engagement in decision-making to increase: (1) understanding about how a decisions is developed, (2) trust in decision-makers, and (3) the quality of decisions and acceptability of decisions (2, 3). Furthermore, to provide more context as to how the reasons for engagement in the survey were identified, quotes following from a former study by Kemper et al. (2022) are stated in Table S2.1. In this study, focus groups with Dutch citizens were executed regarding their views on public engagement in decision-making in COVID-19 management on a broad, public level. In the survey, these views were specified to an individual level, therefore some specific individual reasons are not supported by quotes.

| S2.1 Quotes from a study by Kemper et al. (2022) that executed focus groups regarding the same topic as the survey. The presented quotes are used in the setup of Theme 1 of the survey – reasons to engage. | | |
| --- | --- | --- |
| **Reasons to engage ( used in survey)** | **Supporting quote** | **Age category of participants of focus group** |
| Better understanding of how NPI was developed | ‘’If you have the possibility to think along, you will receive more information, so you will know more and there is more of a discussion in place.’’ | Age 46-64 |
| Decrease overall anxiety about COVID-19 epidemic | ‘’And I also think that people will be left with fewer question, fewer insecurities, if you engage people more. . . if you engage citizens, we will also know what the government is struggling with, and we can think along. So I guess it will not only help solidarity and support, but I think there will be less panic. There are people here who do not even dare to come outside right now. If you engage the people a little bit more, they will receive more information about the situation or about different perspectives, and this I think will decrease panic in society.’’ | Age 18-45 |
| Increase trust in government | ‘’As a citizen, you feel a need to be able to trust the persons who are crucial in such a crisis, such as policy and decision-makers. Well, this is only possible if they make it clear why they made a decision and what they would do differently next time. Transparency builds trusts, and trust during a crisis is very important.’’ | Age 65+ |
| Increase acceptability of NPI | ‘’Sometimes it is difficult to accept decisions that are made by only a single organization. If there would be more of a dialogue in place, or at least create a feeling of being listened to, I think people would accept decisions better.’’ | Age 18-35 |
| Increase quality of NPI | ‘’Well with feedback you can improve the information from the government. You can figure out what you should add or drop by asking other sources.’’ | Age 46-64 |

A similar process was followed for the setup of the reasons not to engage in the survey; these were known from general literature on public engagement in healthcare or policy decision-making and the findings of Kemper et al. (2022), see table S2.2

| S2.2 Supporting quotes from Kemper et al. (2022), used in the setup of Theme 1 – reasons not to engage. | | |
| --- | --- | --- |
| **Reasons not to engage (used in survey)** | **Supporting quote** | **Age category of participants of focus group** |
| Too little knowledge about NPI | ‘’There are a lot of unknowledgeable people. I think that almost every citizen will make decisions based on their gut feeling, without having any knowledge about the crisis. This is very risky.’’ | Age 46-64 |
| No need to engage in general | ‘’I think that currently, some modes of engagement are already executed because that of the representative democracy in our country.’’ | Age 65+ |

**Theme 2 – mode of engagement**


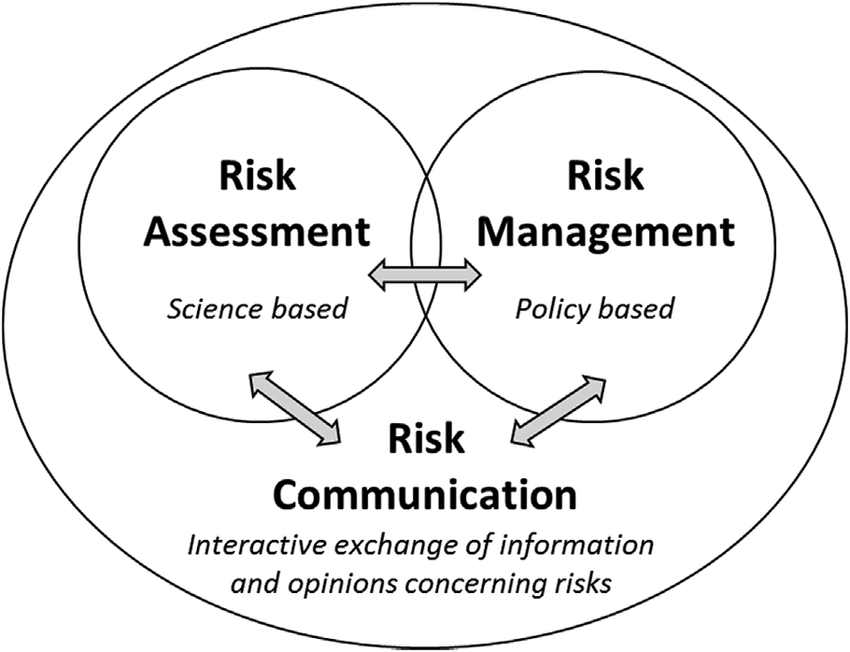

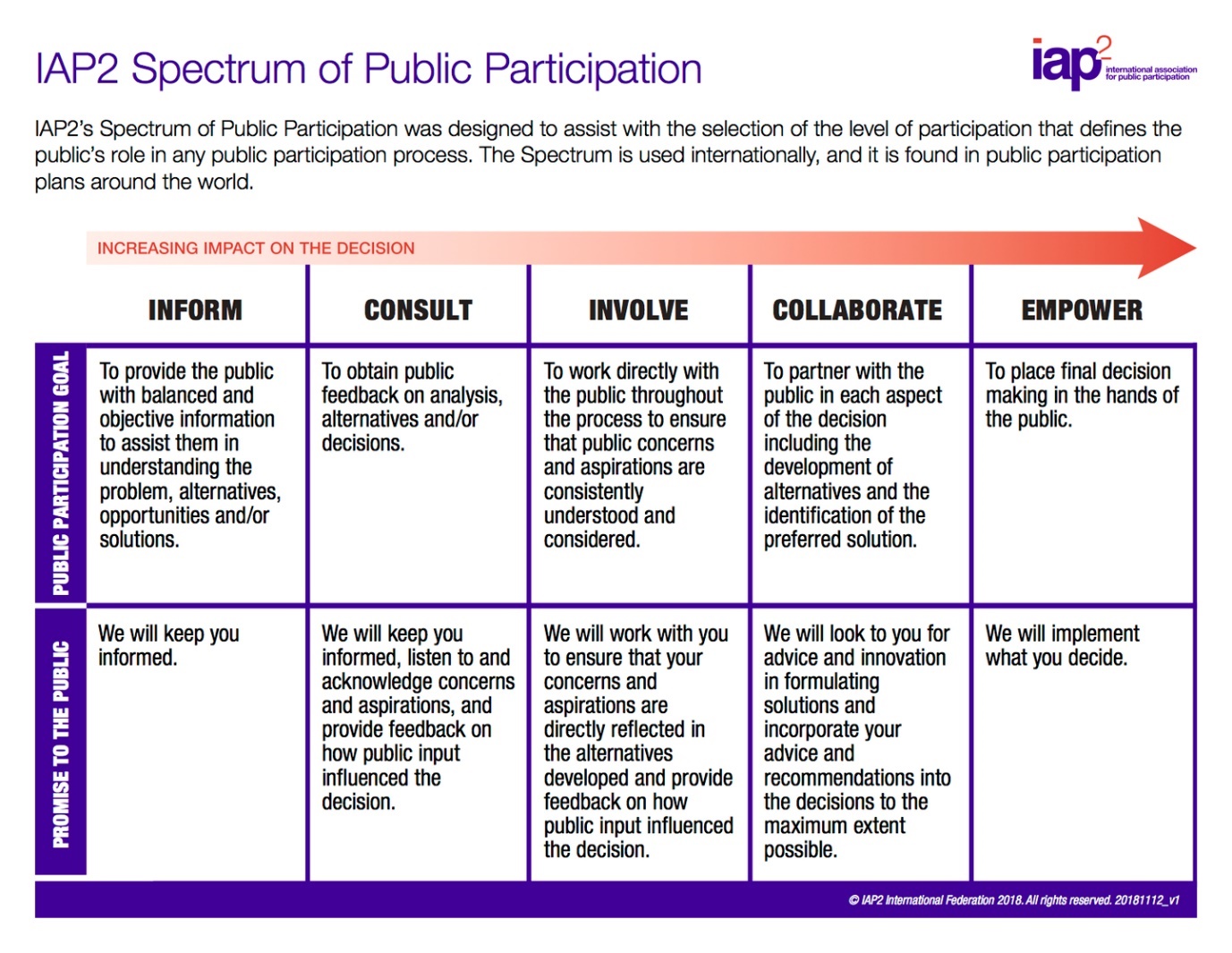
For the second theme, the most suitable modes of engagement, the IAP2 Spectrum of Public Participation was used. This Framework comprises five possible modes of public engagement, depending on the type of interaction and power: *inform*, *consult*, *involve*, *collaborate* and *empower*. The modes of engagement range from providing the public with balanced and objective information (inform), to placing final decision-making in the hands of the public (*empower*), see figure 1. Only the necessary information to clearly explain every mode of engagement was used from the IAP2 framework (which can be found in the original survey). It was stressed that respondents would never be engaged as an individual, but always as a group of citizens, as this could be a relevant consideration in how respondents want to engage (4).

Figure 1.The IAP2 spectrum of public participation, displaying five levels of engagement with their goals and promise to the public. With each level, the impact on decision-making increases. From the International Association for Public Participation (IAP2), 2018.

**Theme 3 – phase in decision-making process**

Theme 3 focused on the phases in the decision-making process regarding the NPIs. These phases were based on the Risk Analysis Framework, displayed in Figure 2 (5). When an outbreak occurs, this framework is used to develop an estimate of the risk of the outbreak to human health and safety (Risk assessment); to identify control measures (Risk management) and to communicate with stakeholders about the risk and measures (Risk communication) (1). The steps in Risk assessment were presented in the survey as assessing the severity of the outbreak situation and determining the effect of the NPI. The steps in Risk management were presented in the survey as making trade-offs between interest and the manner of implementing the NPI. Finally, Risk communication was presented in the survey as the manner of communication regarding the NPI.

Figure 2. The risk analysis framework, adapted from the WHO, displays the three aspects of outbreak management and their interaction.


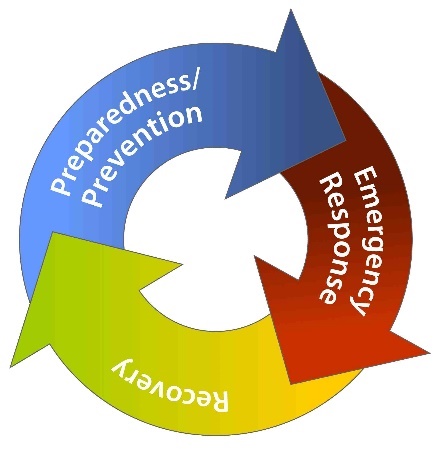
**Theme 4 – timing of engagement**

For timing of engagement, the Emergency Management Cycle was used as the foundation. As displayed in Figure 3, three phases can be distinguished; preparedness, response and recovery. This information was extrapolated into the three answer categories regarding the best timing for engagement; before, during or after the outbreak (6).

**Theme 5 – responsibilities**

Figure 3. The emergency management cycle, adapted from the WHO, displaying three stages in a crisis.

Respondents were asked *who* they thought should have been engaged, who has the responsibility for decision-making, and to what extent contributions of the public should be mandatorily incorporated into decision-making. The questions regarding the mandatory incorporation of the contributions of citizens and the amount of responsibility for politicians, experts and citizens were both based on the findings of Kemper et al. (2022). The answer option categories for whom to engage were based on general literature regarding recruitment methods for public engagement (7, 8). In this literature, self-selection and using a representative sample, for example, are well-known methods to recruit participants.

**References**

1. Kemper S, Kupper F, Kengne Kamga S, Brabers A, De Jong J, Bongers M, et al. Public engagement in decision-making regarding the management of the COVID-19 epidemic: Views and expectations of the 'publics'. Health Expect. 2022.

2. Blumer H. Public Opinion and Public Opinion Polling. American Sociological Review. 1948;13(5):542-9.

3. Rowe G, Frewer L. A Typology of Public Engagement Mechanisms. Science, technology and human values 30 (2005) 2. 2005;30.0.

4. International Association for Public Participation. IAP2’s Public Participation Spectrum (2014). https://cdn.ymaws.com/www.iap2.org/resource/resmgr/pillars/Spectrum_8.5x11_Print.pdf [Accessed Jun 22, 2021].

5. World Health Organization & Food and Agriculture Organization of the United Nations. Risk communication applied to food safety: Handbook. World Health organization, 2016. Available: https:// apps.who. int/ iris/ handle/ 10665/ 250083

6. World Health Organization. Emergency cycle (2022). https://www.who.int/europe/emergencies/emergency-cycle [Accessed Jun 22, 2021].

7. Massie J, Boothe K. Recruiting for engagement in health policy (2022). https://www.engagementinhealthpolicy.ca/blog/recruiting-for-engagement-in-health-policy [Accessed May 22, 2021]

8. Faulkner W, Bynner C. How to Design and Plan Public Engagement Processes: A Handbook. What Works Scotland (2020). 68p.
