# Supplementary material for: Preferences for public engagement in decision-making regarding four COVID-19 non-pharmaceutical interventions in the Netherlands: A survey study
Source: PLoS One. 2023 Oct 5;18(10):e0292119. doi: 10.1371/journal.pone.0292119 (PMC10553365; doi:10.1371/journal.pone.0292119)
Supplement: S3 File — (DOCX) [file pone.0292119.s003.docx]

## Supplementary file 3 – Complete survey

**Survey: Engaging the public in COVID-19 management.**

*The original language of this survey is Dutch, please contact the first author for the original survey in Dutch. Please note, the horizontal line represents a page break in the survey.

Dear participant,

The National Institute for Public Health and Environment and VU University Amsterdam are performing a research about engagement of the public in dealing with the COVID-19 outbreak. How can we use the experiences and ideas of the public to improve the way we deal with the COVID-19 outbreak? Which subjects can the public be engaged in? And in what kind of manner can we do this best? This survey is a part of a bigger research project about the engagement of the public in the management of COVID-19.

The duration of this survey is around 15 minutes. The results will be used for scientific research. Participation in this survey is completely voluntarily. You can stop at any given time. The data will be handled with care and confidentiality, according to the appropriate privacy declaration.

The term *‘’Engagement’’* is used as you having a voice, or participation in decision-making about COVID-19 management. Your opinion, ideas and experiences will be used to design or develop the management of COVID-19.

During this survey, we will be asking you questions about the following non-pharmaceutical interventions (NPIs) that were taken during the epidemic in the Netherlands:

- NPI 1 *(randomly allocated from a pool of 4 NPIs)*

- NPI 2 *(randomly allocated from a pool of 3 NPIs)*

For every NPI, we will explain how it was established and which considerations were taken. In general, this was done in five different steps:

*1. The assessment of the severity of the outbreak situation*

*2. The determination of the effect of the NPI*

*3. Thinking about the trade-offs between interests*

*4. Thinking about how to best implement the NPI*

*5. Thinking about the manner of communication*

First, we will ask **if** you have wanted to be engaged in abovementioned five steps. After that, we will ask you **how** you have wanted to been engaged, **when** would be the best timing to be engaged according to you, **who** should have been engaged, and **reasons why** you did wanted or did not wanted to be engaged. We will ask these questions for every NPI. For both NPIs you have to fill in, we will ask you similar questions. However, your answer can differ, as different considerations come into play. Your work today is important for the role of the public in future crisis situations.

**Routing: if randomization strategy choose The nightly curfew for NPI 1 or NPI 2*

### **The nightly curfew**

On the 23^rd^ of January 2021, a nightly curfew was implemented in the Netherlands. During the implementation, several different considerations were at play, e.g. the effect of the nightly curfew, the need for implementation and how it would be best implemented. We would like to know if you have wanted to be engaged in these considerations in practice, before the actual introduction of the nightly curfew.

As a first step, the severity of the outbreak situation was assessed. A news item stated:

*‘’We are already in a lockdown, but still there are serious concerns: the currently implemented NPIs have too little effect. The number of infections are slowly decreases. However because of the more contagious British and South-African variant of the virus, we are fearing an increase of infections. Experts are saying that we need a more strict policy, as they are fearing a third wave.’’*

1. Would you have wanted to be engaged in assessing the severity of the outbreak situation before introducing the nightly curfew?

Certainly not ○ ○ ○ ○ ○ most certainly

As a second step, the effect of the NPI on the outbreak situation was determined. The virus is transmissible from human to human. Due to this, less transmissions occur when people are less in contact with one another. A news article stated:

*‘’Last week it was told that no calculations can be made about the effect of the curfew, due to lack of data. Nevertheless, experts still think that a nightly curfew could have an effect: ‘’The curfew is a severe NPI, but still a way to reduce contact, especially among young people.’’*

1. Would you have wanted to be engaged in determining the effect of the nightly curfew?

Certainly not ○ ○ ○ ○ ○ most certainly

In the next step, trade-offs were made between the interests regarding the nightly curfew, to make a decision. Interests were e.g. the high severity of the outbreak situation and the fact that there were not many other NPIs to take.

*‘’Most transmission take place during gatherings of families and friends, which will decrease because of the nightly curfew. However, there are a few disadvantages as this NPI is difficult to enforce, and it highly intervenes with the freedom of citizens.’’*

1. Would you have wanted to be engaged in thinking about the trade-offs between the interests regarding the nightly curfew?

Certainly not ○ ○ ○ ○ ○ most certainly

Besides, the practicability of the nightly curfew was taken in consideration. People who had to leave their house could ask for a declaration from the government e.g. for work or an exam. People were also allowed to walk their pets outside during the curfew.

1. Would you have wanted to be engaged in thinking about how to best implement the nightly curfew?

Certainly not ○ ○ ○ ○ ○ most certainly

During the last step, the manner of communication about the nightly curfew was considered. How could this NPI be best communicated to the public, and via which channels? You can think about press conferences, apps and newspapers.

1. Would you have wanted to be engaged in thinking about the manner of communication regarding the nightly curfew?

Certainly not ○ ○ ○ ○ ○ most certainly

1. Would you like to give an explanation of the answers you gave to the previous questions?

…………………………………………………………………………………….…………………………………………………………………………………….………………..… ...………………………………………………………………….…………………………………………………………………………………….………………………………..…………………………………………………………………………………………………………………………………….……………………………………………………………

Below you are presented with 5 modes of engagement. Please read these 5 modes carefully.

1. *Inform:* You will receive all information about the considerations regarding the nightly curfew, and how the final decision is made.
2. *Feedback*: You will give your opinion regarding certain questions or problems about the nightly curfew e.g. the manner of communication. You opinion will be taken under consideration by decision-makers, but they are not obliged to use it in the final decision.
3. *Advice*: Your advice is asked regarding all steps in the decision-making process, and will be certainly used in the final decision.
4. *Collaborate*: You will collaborate with the government regarding all decisions about the nightly curfew. For example you will consider all the different interests at play and the manner of communication. You will make the final decision together with the government.
5. *Power*: You have the final decision-making power regarding the nightly curfew and how this will be implemented in the Netherlands. The government will support you.
6. Rank the 5 modes of engagement in order from least suitable (1) to most suitable (5) according to how you would have liked to be engaged in the nightly curfew. Please note, you will never be engaged as an individual, engagement will always be done with groups of citizens.

1=

2=

3=

4=

5=

1. Would you like to give an explanation of the answers you gave to the questions?

…………………………………………………………………………………….…………………………………………………………………………………….………………..… ...………………………………………………………………….…………………………………………………………………………………….………………………………..…………………………………………………………………………………………………………………………………….……………………………………………………………

1. When do you think is the best time to be engaged in the nightly curfew? Multiple answers possible.
   - Before the COVID-19 outbreak. We did not know if the nightly curfew would ever be implemented, but there would be clarity about the opinions of citizens about the curfew.
   - During the COVID-19 outbreak, when it became clear that the nightly curfew would be necessary.
   - After the COVID-19 outbreak, during an evaluation. This information can be used for future epidemics.
   - Other, namely …..
   - Never.
2. Suppose that you were engaged in the nightly curfew. To what extent do you think that your contributions and that of other citizens should be incorporated in the final decision about the nightly curfew? Choose the answer that best fits you.

1 = incorporation of your contributions without any obligations, 5 = mandatory incorporations of your contributions. In all answers, the contributions of you and other citizens will always be seriously considered.

1 ○ 2 ○ 3 ○ 4 ○ 5 ○

1. Who do you think should have been engaged in the nightly curfew? Multiple answers possible.

- All citizens should have been engaged.
- Citizens who wanted it themselves, should have been able to sign up for engagement.
- Certain organisations or corporations should have been engaged, that had a lot to do with the nightly curfew. For example associations for restaurants, hotels and healthcare organisations.
- Certain persons representing interest groups within society, for example the leader of a youth organisation.
- A representative sample of the Dutch population, chosen based on certain demographic characteristics such as age and gender.
- Other, namely …..
- None of the above.

1. What amount of responsibility should the following groups have regarding decision-making about the nightly curfew?

|  | No responsibility at all | A little responsibility | Average responsibility | A lot of responsibility | All responsibility |
| --- | --- | --- | --- | --- | --- |
| Politicians in the government |  |  |  |  |  |
| Experts in epidemics |  |  |  |  |  |
| Citizens |  |  |  |  |  |

1. In general, would you have wanted to be engaged in the decisions regarding the nightly curfew?

- Yes
- No
- I don’t know / neutral

**Routing: only if respondent has answered yes to question 13.*

1. We would like to know your reasoning as to why you answered that you **did** wanted to be engaged in the decisions regarding the nightly curfew in practice. Indicate whether the following statements are true for you or not.

|  | Not true | Not really true | Neutral | A little true | True |
| --- | --- | --- | --- | --- | --- |
| I did wanted to be engaged because it would help me better understand how the nightly curfew was developed. |  |  |  |  |  |
| I did wanted to be engaged because it would decrease the overall anxiety that I have about the COVID-19 epidemic. |  |  |  |  |  |
| I did wanted to be engaged because it would increase my trust in the government. |  |  |  |  |  |
| I did wanted to be engaged because it would increase my adherence to the nightly curfew. |  |  |  |  |  |
| I did wanted to be engaged because it would increase the quality of the nightly curfew overall. |  |  |  |  |  |

1. Besides abovementioned reasons, there may be other reasons that you did wanted to be engaged in the nightly curfew in practice. If so, please enter this below. If not, please click not applicable.

…………………………………………………………………………………….…………………………………………………………………………………….………………..… ...………………………………………………………………….…………………………………………………………………………………….………………………………..…………………………………………………………………………………………………………………………………….……………………………………………………………

**Routing: only if respondent has answered no to question 13.*

1. We would like to know your reasoning as to why you answered that you **did not** wanted to be engaged in the decisions regarding the nightly curfew in practie. Indicate whether the following statements are true for you or not.

|  | Not true | Not really true | Neutral | A little true | True |
| --- | --- | --- | --- | --- | --- |
| I did not wanted to be engaged because I have to little knowledge about the nightly curfew. |  |  |  |  |  |
| I did not wanted to be engaged because I have to little time. |  |  |  |  |  |
| I did not wanted to be engaged because I don’t feel the need to. |  |  |  |  |  |
| I did not wanted to be engaged because the nightly curfew didn’t affect me directly. |  |  |  |  |  |

1. Besides abovementioned reasons, there may be other reasons that you did not wanted to be engaged in the nightly curfew in practice. If so, please enter this below. If not, please click not applicable.

…………………………………………………………………………………….…………………………………………………………………………………….………………..… ...………………………………………………………………….…………………………………………………………………………………….………………………………..…………………………………………………………………………………………………………………………………….……………………………………………………………

**Routing: if randomization strategy choose Closure of elementary & high schools and daycares for NPI 1 or NPI 2*

### **Closure of elementary & high schools and daycares**

From 16^th^ of March until 6^th^ of April 2020, during the first wave of the COVID-19 epidemic, the decision was made to close elementary and high schools and daycares. During the implementation, several different considerations were at play. We would like to know if you have wanted to be engaged in these considerations in practice, before the actual introduction of the closure.

As a first step, the severity of the outbreak situation was assessed. A news item from March 2020 stated:

*‘’As of yesterday, eight people died due to COVID-19 in the Netherlands. In total, twenty people have lost their lives because of it. Besides, there are 176 new positive tests, which makes a total of 1135 infected people in the Netherlands.’’*

1. Would you have wanted to be engaged in assessing the severity of the outbreak situation before introducing the closure?

Certainly not ○ ○ ○ ○ ○ most certainly

As a second step, the effect of the NPI on the outbreak situation was determined. A news item stated:

*‘’A pediatrician who studied COVID-19 infections in children concluded that there were never any scientific arguments for the closure of schools. She indicates that closing schools only helps to reduce deaths by 2% to 4%. Social distancing contributed to a 44% reduction.’’*

1. Would you have wanted to be engaged in determining the effect of the closure of schools and daycares?

Certainly not ○ ○ ○ ○ ○ most certainly

In the next step, trade-offs were made between the interests regarding the closure, to make a decision. Furthermore, the practicability of the closure of schools and daycares was taken in consideration.

*‘’Due to the many sick leaves – staying home with any COVID-related symptoms – teachers were no longer able to work. The concern of teachers and parents also played a role in the decision to close the schools.’’*

*‘’Closing the schools however, does have a downside. You deny children their education, you could create difficult situations at home, and you keep healthcare staff from their jobs in order to take care of their children. Since the closure of school, signs of domestic violence has increased.’’*

1. Would you have wanted to be engaged in thinking about the trade-offs between the interests regarding the closure?

Certainly not ○ ○ ○ ○ ○ most certainly

1. Would you have wanted to be engaged in thinking about how to best implement the closure?

Certainly not ○ ○ ○ ○ ○ most certainly

During the last step, the manner of communication about the closure was considered. How could this NPI be best communicated to the public, and via which channels? You can think about press conferences, apps and newspapers.

1. Would you have wanted to be engaged in thinking about the manner of communication regarding the closure?

Certainly not ○ ○ ○ ○ ○ most certainly

1. Would you like to give an explanation of the answers you gave to the previous questions?

…………………………………………………………………………………….…………………………………………………………………………………….………………..… ...………………………………………………………………….…………………………………………………………………………………….………………………………..…………………………………………………………………………………………………………………………………….……………………………………………………………

Below you are presented with 5 modes of engagement. Please read these 5 modes carefully.

1. *Inform:* You will receive all information about the considerations regarding the closure of schools and daycares, and how the final decision is made.
2. *Feedback*: You will give your opinion regarding certain questions or problems about the closure e.g. the manner of communication. You opinion will be taken under consideration by decision-makers, but they are not obliged to use it in the final decision.
3. *Advice*: Your advice is asked regarding all steps in the decision-making process, and will be certainly used in the final decision.
4. *Collaborate*: You will collaborate with the government regarding all decisions about the closure. For example you will consider all the different interests at play and the manner of communication. You will make the final decision together with the government.
5. *Power*: You have the final decision-making power regarding the closure and how this will be implemented in the Netherlands. The government will support you.
6. Rank the 5 modes of engagement in order from least suitable (1) to most suitable (5) according to how you would have liked to be engaged in the closure of schools and daycares. Please note, you will never be engaged as an individual, engagement will always be done with groups of citizens.

1=

2=

3=

4=

5=

1. Would you like to give an explanation of the answers you gave to the questions?

…………………………………………………………………………………….…………………………………………………………………………………….………………..… ...………………………………………………………………….…………………………………………………………………………………….………………………………..…………………………………………………………………………………………………………………………………….……………………………………………………………

1. When do you think is the best time to be engaged in the closure of schools and daycare? Multiple answers possible.
   - Before the COVID-19 outbreak. We did not know if the closure would ever be implemented, but there would be clarity about the opinions of citizens about the closure.
   - During the COVID-19 outbreak, when it became clear that the closure would be necessary.
   - After the COVID-19 outbreak, during an evaluation. This information can be used for future epidemics.
   - Other, namely …..
   - Never.
2. Suppose that you were engaged in the closure of schools and daycares. To what extent do you think that your contributions and that of other citizens should be incorporated in the final decision about the closure? Choose the answer that best fits you.

1 = incorporation of your contributions without any obligations, 5 = mandatory incorporations of your contributions. In all answers, the contributions of you and other citizens will always be seriously considered.

1 ○ 2 ○ 3 ○ 4 ○ 5 ○

1. Who do you think should have been engaged in the closure of schools and daycares? Multiple answers possible.

- All citizens should have been engaged.
- Citizens who wanted it themselves, should have been able to sign up for engagement.
- Certain organisations or corporations should have been engaged, that had a lot to do with the closure. For example associations schools or daycares.
- Certain persons representing interest groups within society, for example the leader of a parent organisation.
- A representative sample of the Dutch population, chosen based on certain demographic characteristics such as age and gender.
- Other, namely …..
- None of the above.

1. What amount of responsibility should the following groups have regarding decision-making about the closure of schools and daycares?

|  | No responsibility at all | A little responsibility | Average responsibility | A lot of responsibility | All responsibility |
| --- | --- | --- | --- | --- | --- |
| Politicians in the government |  |  |  |  |  |
| Experts in epidemics |  |  |  |  |  |
| Citizens |  |  |  |  |  |

1. In general, would you have wanted to be engaged in the decisions regarding the closure?

- Yes
- No
- I don’t know / neutral

**Routing: only if respondent has answered yes to question 13.*

1. We would like to know your reasoning as to why you answered that you **did** wanted to be engaged in the decisions regarding the closure in practice. Indicate whether the following statements are true for you or not.

|  | Not true | Not really true | Neutral | A little true | True |
| --- | --- | --- | --- | --- | --- |
| I did wanted to be engaged because it would help me better understand how the closure was developed. |  |  |  |  |  |
| I did wanted to be engaged because it would decrease the overall anxiety that I have about the COVID-19 epidemic. |  |  |  |  |  |
| I did wanted to be engaged because it would increase my trust in the government. |  |  |  |  |  |
| I did wanted to be engaged because it would increase my acceptance of the closure. |  |  |  |  |  |
| I did wanted to be engaged because it would increase the quality of the closure overall. |  |  |  |  |  |

1. Besides abovementioned reasons, there may be other reasons that you did wanted to be engaged in the closure in practice. If so, please enter this below. If not, please click not applicable.

…………………………………………………………………………………….…………………………………………………………………………………….………………..… ...………………………………………………………………….…………………………………………………………………………………….………………………………..…………………………………………………………………………………………………………………………………….……………………………………………………………

**Routing: only if respondent has answered no to question 13.*

1. We would like to know your reasoning as to why you answered that you **did not** wanted to be engaged in the decisions regarding the closure in practice. Indicate whether the following statements are true for you or not.

|  | Not true | Not really true | Neutral | A little true | True |
| --- | --- | --- | --- | --- | --- |
| I did not wanted to be engaged because I have to little knowledge about the closure. |  |  |  |  |  |
| I did not wanted to be engaged because I have to little time. |  |  |  |  |  |
| I did not wanted to be engaged because I don’t feel the need to. |  |  |  |  |  |
| I did not wanted to be engaged because the closure didn’t affect me directly. |  |  |  |  |  |

1. Besides abovementioned reasons, there may be other reasons that you did not wanted to be engaged in the closure in practice. If so, please enter this below. If not, please click not applicable.

…………………………………………………………………………………….…………………………………………………………………………………….………………..… ...………………………………………………………………….…………………………………………………………………………………….………………………………..…………………………………………………………………………………………………………………………………….……………………………………………………………

**Routing: if randomization strategy choose Covid entry pass for events for NPI 1 or NPI 2.*

### **COVID entry pass for events**

At the start of July 2021, there was a short period of time in which mandatory COVID entry passes for events were used. Due to these passes, social distancing was no longer necessary and the number of visitors could be increased. The COVID entry pass could consist of a negative test result, a vaccination certificate or a recovery certificate. During the implementation, several different considerations were at play. We would like to know if you have wanted to be engaged in these considerations in practice, before the actual introduction of the entry pass.

As a first step, the severity of the outbreak situation was assessed. A news item from June 2021 stated:

*‘’The vaccination rate is increasing rapidly, up to more than 13 million. Almost 5 million citizens are now fully vaccinated. Starting tomorrow, also 18-year old’s can make a vaccination appointment. Which means that mid-July, everyone who wants to, can be vaccinated. This is reflected in the infection rates and the number of hospital admissions. These numbers are rapidly decreasing.’’*

1. Would you have wanted to be engaged in assessing the severity of the outbreak situation before introducing the COVID entry pass?

Certainly not ○ ○ ○ ○ ○ most certainly

As a second step, the effect of the NPI on the outbreak situation was determined. The evidence showed that there is a low chance of transmission by using this pass. A report stated:

*‘’Since the start of this year, experiments with testing have been executed to make events safer. By means of testing for events, people who are infected will be identified. These people will not gain access, which increases the safety of the event.’’*

1. Would you have wanted to be engaged in determining the effect of the entry pass?

Certainly not ○ ○ ○ ○ ○ most certainly

In the next step, trade-offs were made between the interests regarding the entry pass, to make a decision. By implementing the entry pass, people could have more freedom. A news item stated:

*‘’Most of the people who are against the use of the COVID entry pass understand the idea behind it, but still see the disadvantages. Some fear that the COVID entry pass will lead to some sort of twisted vaccination obligation. Most people against it fear that it will lead to inequality in society.’’*

*By using the COVID entry pass, society can open again, even if the virus is not yet completely controlled. This makes it possible to visit theaters, sport events and events in general.’’*

*‘’It would be good for our economy, and also for the well-being of people.’’*

1. Would you have wanted to be engaged in thinking about the trade-offs between the interests regarding the entry pass?

Certainly not ○ ○ ○ ○ ○ most certainly

Besides, the practicability of the entry pass was taken in consideration. A digital application was developed for the entry pass: The COVID-Check-app. Besides, the entry pass itself was free.

1. Would you have wanted to be engaged in thinking about how to best implement the entry pass?

Certainly not ○ ○ ○ ○ ○ most certainly

During the last step, the manner of communication about the entry pass was considered. How could this NPI be best communicated to the public, and via which channels? You can think about press conferences, apps and newspapers.

1. Would you have wanted to be engaged in thinking about the manner of communication regarding the entry pass?

Certainly not ○ ○ ○ ○ ○ most certainly

1. Would you like to give an explanation of the answers you gave to the previous questions?

…………………………………………………………………………………….…………………………………………………………………………………….………………..… ...………………………………………………………………….…………………………………………………………………………………….………………………………..…………………………………………………………………………………………………………………………………….……………………………………………………………

Below you are presented with 5 modes of engagement. Please read these 5 modes carefully.

1. *Inform:* You will receive all information about the considerations regarding the entry pass, and how the final decision is made.
2. *Feedback*: You will give your opinion regarding certain questions or problems about the entry pass e.g. the manner of communication. You opinion will be taken under consideration by decision-makers, but they are not obliged to use it in the final decision.
3. *Advice*: Your advice is asked regarding all steps in the decision-making process, and will be certainly used in the final decision.
4. *Collaborate*: You will collaborate with the government regarding all decisions about the entry pass. For example you will consider all the different interests at play and the manner of communication. You will make the final decision together with the government.
5. *Power*: You have the final decision-making power regarding the entry pass and how this will be implemented in the Netherlands. The government will support you.
6. Rank the 5 modes of engagement in order from least suitable (1) to most suitable (5) according to how you would have liked to be engaged in the entry pass. Please note, you will never be engaged as an individual, engagement will always be done with groups of citizens.

1=

2=

3=

4=

5=

1. Would you like to give an explanation of the answers you gave to the questions?

…………………………………………………………………………………….…………………………………………………………………………………….………………..… ...………………………………………………………………….…………………………………………………………………………………….………………………………..…………………………………………………………………………………………………………………………………….……………………………………………………………

1. When do you think is the best time to be engaged in the COVID entry pass? Multiple answers possible.
   - Before the COVID-19 outbreak. We did not know if the entry pass would ever be implemented, but there would be clarity about the opinions of citizens about the pass.
   - During the COVID-19 outbreak, when it became clear that the entry pass would be necessary.
   - After the COVID-19 outbreak, during an evaluation. This information can be used for future epidemics.
   - Other, namely …..
   - Never.
2. Suppose that you were engaged in the entry pass. To what extent do you think that your contributions and that of other citizens should be incorporated in the final decision about the pass? Choose the answer that best fits you.

1 = incorporation of your contributions without any obligations, 5 = mandatory incorporations of your contributions. In all answers, the contributions of you and other citizens will always be seriously considered.

1 ○ 2 ○ 3 ○ 4 ○ 5 ○

1. Who do you think should have been engaged in the entry pass? Multiple answers possible.

- All citizens should have been engaged.
- Citizens who wanted it themselves, should have been able to sign up for engagement.
- Certain organisations or corporations should have been engaged, that had a lot to do with the entry pass. For example associations for restaurants, hotels and events.
- Certain persons representing interest groups within society, for example the leader of a youth organisation.
- A representative sample of the Dutch population, chosen based on certain demographic characteristics such as age and gender.
- Other, namely …..
- None of the above.

1. What amount of responsibility should the following groups have regarding decision-making about the entry pass?

|  | No responsibility at all | A little responsibility | Average responsibility | A lot of responsibility | All responsibility |
| --- | --- | --- | --- | --- | --- |
| Politicians in the government |  |  |  |  |  |
| Experts in epidemics |  |  |  |  |  |
| Citizens |  |  |  |  |  |

1. In general, would you have wanted to be engaged in the decisions regarding the entry pass?

- Yes
- No
- I don’t know / neutral

**Routing: only if respondent has answered yes to question 13.*

1. We would like to know your reasoning as to why you answered that you **did** wanted to be engaged in the decisions regarding the entry pass in practice. Indicate whether the following statements are true for you or not.

|  | Not true | Not really true | Neutral | A little true | True |
| --- | --- | --- | --- | --- | --- |
| I did wanted to be engaged because it would help me better understand how the entry pass was developed. |  |  |  |  |  |
| I did wanted to be engaged because it would decrease the overall anxiety that I have about the COVID-19 epidemic. |  |  |  |  |  |
| I did wanted to be engaged because it would increase my trust in the government. |  |  |  |  |  |
| I did wanted to be engaged because it would increase my acceptance of the entry pass. |  |  |  |  |  |
| I did wanted to be engaged because it would increase the quality of the entry pass overall. |  |  |  |  |  |

1. Besides abovementioned reasons, there may be other reasons that you did wanted to be engaged in the entry pass in practice. If so, please enter this below. If not, please click not applicable.

…………………………………………………………………………………….…………………………………………………………………………………….………………..… ...………………………………………………………………….…………………………………………………………………………………….………………………………..…………………………………………………………………………………………………………………………………….……………………………………………………………

**Routing: only if respondent has answered no to question 13.*

1. We would like to know your reasoning as to why you answered that you **did not** wanted to be engaged in the decisions regarding the entry pass in practice. Indicate whether the following statements are true for you or not.

|  | Not true | Not really true | Neutral | A little true | True |
| --- | --- | --- | --- | --- | --- |
| I did not wanted to be engaged because I have to little knowledge about the entry pass. |  |  |  |  |  |
| I did not wanted to be engaged because I have to little time. |  |  |  |  |  |
| I did not wanted to be engaged because I don’t feel the need to. |  |  |  |  |  |
| I did not wanted to be engaged because the entry pass didn’t affect me directly. |  |  |  |  |  |

1. Besides abovementioned reasons, there may be other reasons that you did not wanted to be engaged in the entry pass in practice. If so, please enter this below. If not, please click not applicable.

…………………………………………………………………………………….…………………………………………………………………………………….………………..… ...………………………………………………………………….…………………………………………………………………………………….………………………………..…………………………………………………………………………………………………………………………………….……………………………………………………………

**Routing: if randomization strategy choose 1.5meter social distancing for NPI 1 or NPI 2*

### **1.5meter social distancing**

Since March 2020, a number of basic restriction measures are in place in the Netherlands. The 1.5meter social distancing was one of them. Everyone was asked to implement social distancing. During the implementation, several different considerations were at play. We would like to know if you have wanted to be engaged in these considerations in practice, before the actual introduction of the 1.5meter social distancing.

As a first step, the severity of the outbreak situation was assessed. In March 2020, the COVID-19 outbreak just started in the Netherlands. The number of infections was increases, and together with 1.5metre social distancing, other restriction measures were taken.

1. Would you have wanted to be engaged in assessing the severity of the outbreak situation before introducing the 1.5m social distance?

Certainly not ○ ○ ○ ○ ○ most certainly

As a second step, the effect of the NPI on the outbreak situation was determined. A news item stated:

*‘’If there is a virus that spreads through coughing and spitting, keep your distance. At least one meter, according to the WHO. Two meters, is the agreement in Spain. 1.8 meter, according to experts in the US.’’*

*‘’It is clear that social distancing works. Respiratory viruses such as SARS-Cov-II mainly infect people who have been in close contact with one another, e.g. family members or colleagues.*

1. Would you have wanted to be engaged in determining the effect of the 1.5m social distance?

Certainly not ○ ○ ○ ○ ○ most certainly

In the next step, trade-offs were made between the interests regarding the 1.5m social distance, to make a decision. A news item stated:

*‘’During the period that more restriction measures were necessary in the Netherlands, the choice was made to implement 1.5meter social distancing. On the one hand, the WHO advised at least one meter, and the Dutch expert panel advised two meters. For clear communication, and because not everyone has immediate access to measuring tape, we therefore advice to use two arm’s lengths. Which comes down to about 1.5meters, and is easy to manage.’’*

So, among other things, the outbreak situation, expert knowledge, and the practicability of executing the 1.5m social distance were taken into account.

1. Would you have wanted to be engaged in thinking about the trade-offs between the interests regarding the 1.5m social distance?

Certainly not ○ ○ ○ ○ ○ most certainly

1. Would you have wanted to be engaged in thinking about how to best implement the 1.5m social distance?

Certainly not ○ ○ ○ ○ ○ most certainly

During the last step, the manner of communication about the 1.5m social distance was considered. How could this NPI be best communicated to the public, and via which channels? You can think about press conferences, apps and newspapers.

1. Would you have wanted to be engaged in thinking about the manner of communication regarding the 1.5m social distance?

Certainly not ○ ○ ○ ○ ○ most certainly

1. Would you like to give an explanation of the answers you gave to the previous questions?

…………………………………………………………………………………….…………………………………………………………………………………….………………..… ...………………………………………………………………….…………………………………………………………………………………….………………………………..…………………………………………………………………………………………………………………………………….……………………………………………………………

Below you are presented with 5 modes of engagement. Please read these 5 modes carefully.

1. *Inform:* You will receive all information about the considerations regarding the 1.5m social distance, and how the final decision is made.
2. *Feedback*: You will give your opinion regarding certain questions or problems about the 1.5m e.g. the manner of communication. You opinion will be taken under consideration by decision-makers, but they are not obliged to use it in the final decision.
3. *Advice*: Your advice is asked regarding all steps in the decision-making process, and will be certainly used in the final decision.
4. *Collaborate*: You will collaborate with the government regarding all decisions about the 1.5m. For example you will consider all the different interests at play and the manner of communication. You will make the final decision together with the government.
5. *Power*: You have the final decision-making power regarding the 1.5m and how this will be implemented in the Netherlands. The government will support you.
6. Rank the 5 modes of engagement in order from least suitable (1) to most suitable (5) according to how you would have liked to be engaged in the 1.5m. Please note, you will never be engaged as an individual, engagement will always be done with groups of citizens.

1=

2=

3=

4=

5=

1. Would you like to give an explanation of the answers you gave to the questions?

…………………………………………………………………………………….…………………………………………………………………………………….………………..… ...………………………………………………………………….…………………………………………………………………………………….………………………………..…………………………………………………………………………………………………………………………………….……………………………………………………………

1. When do you think is the best time to be engaged in the 1.5m social distance? Multiple answers possible.
   - Before the COVID-19 outbreak. We did not know if the 1.5m would ever be implemented, but there would be clarity about the opinions of citizens about the 1.5m.
   - During the COVID-19 outbreak, when it became clear that the 1.5m would be necessary.
   - After the COVID-19 outbreak, during an evaluation. This information can be used for future epidemics.
   - Other, namely …..
   - Never.
2. Suppose that you were engaged in the 1.5m social distance. To what extent do you think that your contributions and that of other citizens should be incorporated in the final decision about the 1.5m? Choose the answer that best fits you.

1 = incorporation of your contributions without any obligations, 5 = mandatory incorporations of your contributions. In all answers, the contributions of you and other citizens will always be seriously considered.

1 ○ 2 ○ 3 ○ 4 ○ 5 ○

1. Who do you think should have been engaged in the 1.5m social distance? Multiple answers possible.

- All citizens should have been engaged.
- Citizens who wanted it themselves, should have been able to sign up for engagement.
- Certain organisations or corporations should have been engaged, that had a lot to do with the 1.5m.
- Certain persons representing interest groups within society, for example the leader of a youth organisation.
- A representative sample of the Dutch population, chosen based on certain demographic characteristics such as age and gender.
- Other, namely …..
- None of the above.

1. What amount of responsibility should the following groups have regarding decision-making about the 1.5m?

|  | No responsibility at all | A little responsibility | Average responsibility | A lot of responsibility | All responsibility |
| --- | --- | --- | --- | --- | --- |
| Politicians in the government |  |  |  |  |  |
| Experts in epidemics |  |  |  |  |  |
| Citizens |  |  |  |  |  |

1. In general, would you have wanted to be engaged in the decisions regarding the 1.5m social distance?

- Yes
- No
- I don’t know / neutral

**Routing: only if respondent has answered yes to question 13.*

1. We would like to know your reasoning as to why you answered that you **did** wanted to be engaged in the decisions regarding the 1.5m social distance in practice. Indicate whether the following statements are true for you or not.

|  | Not true | Not really true | Neutral | A little true | True |
| --- | --- | --- | --- | --- | --- |
| I did wanted to be engaged because it would help me better understand how the 1.5m social distance was developed. |  |  |  |  |  |
| I did wanted to be engaged because it would decrease the overall anxiety that I have about the COVID-19 epidemic. |  |  |  |  |  |
| I did wanted to be engaged because it would increase my trust in the government. |  |  |  |  |  |
| I did wanted to be engaged because it would increase my adherence of the 1.5m social distance. |  |  |  |  |  |
| I did wanted to be engaged because it would increase the quality of the 1.5m social distance overall. |  |  |  |  |  |

1. Besides abovementioned reasons, there may be other reasons that you did wanted to be engaged in the 1.5m social distance in practice. If so, please enter this below. If not, please click not applicable.

…………………………………………………………………………………….…………………………………………………………………………………….………………..… ...………………………………………………………………….…………………………………………………………………………………….………………………………..…………………………………………………………………………………………………………………………………….……………………………………………………………

**Routing: only if respondent has answered no to question 13.*

1. We would like to know your reasoning as to why you answered that you **did not** wanted to be engaged in the decisions regarding the 1.5m social distance in practie. Indicate whether the following statements are true for you or not.

|  | Not true | Not really true | Neutral | A little true | True |
| --- | --- | --- | --- | --- | --- |
| I did not wanted to be engaged because I have to little knowledge about the 1.5m social distance. |  |  |  |  |  |
| I did not wanted to be engaged because I have to little time. |  |  |  |  |  |
| I did not wanted to be engaged because I don’t feel the need to. |  |  |  |  |  |
| I did not wanted to be engaged because the 1.5m social distance didn’t affect me directly. |  |  |  |  |  |

1. Besides abovementioned reasons, there may be other reasons that you did not wanted to be engaged in the 1.5m social distance in practice. If so, please enter this below. If not, please click not applicable.

…………………………………………………………………………………….…………………………………………………………………………………….………………..… ...………………………………………………………………….…………………………………………………………………………………….………………………………..…………………………………………………………………………………………………………………………………….……………………………………………………………

**This is the end of the survey. Thank you very much for participation. We wish you a pleasant day.**
