# Supplementary material for: Preferences for public engagement in decision-making regarding four COVID-19 non-pharmaceutical interventions in the Netherlands: A survey study
Source: PLoS One. 2023 Oct 5;18(10):e0292119. doi: 10.1371/journal.pone.0292119 (PMC10553365; doi:10.1371/journal.pone.0292119)
Supplement: S4 File — (DOCX) [file pone.0292119.s004.docx]

## Supplementary file 4 – Results of subgroup analysis

| S4. Results of subgroup analysis. for the likert-scale questions, chi-square tests were executed. for the proportions, mcnemar tests were executed. benjamini-hochberg method was applied per theme, of which the transformed p-values are displayed in the table. per theme, the number of false positives are explained. the fdr rate was set on 5%. significant results are highlighted in grey. | | | | | | | |
| --- | --- | --- | --- | --- | --- | --- | --- |
| Theme | category | NC - CED | NC - CEP | NC – 1.5M | CED - CEP | CED – 1.5M | CEP – 1.5M |
| Desire for engagement | | 0.01 | <0.001 | <0.001 | <0.001 | <0.001 | <0.001 |
| Reasoning DESIRE FOR ENGAGEMENT | Understanding | 0.91 | 0.20 | 0.80 | <0.001 | 0.90 | 0.40 |
|  | Anxiety | 0.25 | 0.06 | 0.96 | 1.00 | 0.79 | 0.79 |
|  | Trust | 0.02 | <0.001 | <0.001 | 0.02 | 0.55 | <0.001 |
|  | Acceptability | <0.001 | <0.001 | 0.71 | 0.02 | 0.04 | 0.04 |
|  | Quality | 0.69 | 0.48 | 0.26 | 0.55 | 0.20 | 0.18 |
| Reasoning NO DESIRE FOR ENGAGEMENT | Lack of knowledge | <0.001 | 0.72 | 0.79 | <0.001 | <0.001 | <0.001 |
|  | Lack of time | 0.01 | 0.59 | 0.78 | 0.02 | 0.04 | 0.07 |
|  | Lack of need | <0.001 | 0.72 | 0.17 | 0.77 | 0.55 | 0.83 |
|  | No direct impact | <0.001 | <0.001 | <0.001 | <0.001 | <0.001 | <0.001 |
| **FDR for theme ‘’Desire for engagement & reasoning’’; typically no more than 1.4 false positives in abovementioned list of 31 rejections (Transformed P-value < 0.05, as FDR rate is set on 5%)** | | | | | | | |
| phases in decision-making process | Situation assessment | 0.01 | 0.06 | <0.001 | <0.001 | <0.001 | <0.001 |
|  | Effect of [NPI] | <0.001 | 0.1 | <0.001 | <0.001 | 0.24 | <0.001 |
|  | Trade-off between interests | <0.001 | 0.24 | <0.001 | <0.001 | <0.001 | <0.001 |
|  | Practicability | <0.001 | 0.01 | <0.001 | 0.38 | 0.13 | <0.001 |
|  | Communication | <0.001 | 0.38 | 0.1 | <0.001 | 0.36 | <0.001 |
| **FDR for theme ‘’Step in decision-making process’’; typically no more than 0.2 false positives in abovementioned list of 21 rejections (Transformed P-value < 0.05, as FDR rate is set on 5%)** | | | | | | | |
| Timing of engagement | Before the outbreak | 0.21 | 0.49 | 0.59 | 0.61 | 0.71 | 0.48 |
|  | During the outbreak | 0.71 | 0.49 | 0.11 | <0.001 | <0.001 | <0.001 |
|  | After the outbreak | 0.67 | 1.03 | 1.00 | 0.71 | 0.19 | 0.18 |
|  | Other | 0.73 | 0.70 | 0.49 | 0.19 | 0.50 | 0.08 |
|  | Never | <0.001 | 0.02 | 0.21 | <0.001 | <0.001 | 0.10 |
| **FDR for theme ‘’Timing of engagement; typically no more than 0.1 false positives in abovementioned list of 7 rejections (Transformed P-value < 0.05, as FDR rate is set on 5%)** | | | | | | | |
| Who | All citizens | 0.01 | 0.90 | 0.01 | 0.01 | <0.001 | <0.001 |
|  | Signing up | 0.04 | 0.58 | 0.06 | <0.001 | 0.19 | 0.14 |
|  | Organizations and companies | <0.001 | <0.001 | <0.001 | 0.01 | <0.001 | <0.001 |
|  | Persons representing interest groups | <0.001 | 0.38 | 0.22 | <0.001 | <0.001 | <0.001 |
|  | Representative sample of Dutch population | <0.001 | 0.20 | 0.92 | <0.001 | <0.001 | 0.08 |
|  | Other | 1.00 | 0.91 | 0.75 | 0.91 | 0.51 | 0.95 |
|  | None | 0.20 | 0.12 | 0.01 | 0.90 | <0.001 | <0.001 |
| Responsibility | Politicians | 0.02 | 0.05 | <0.001 | 0.07 | <0.001 | <0.001 |
|  | Experts | 0.93 | <0.001 | <0.001 | 0.35 | <0.001 | <0.001 |
|  | Citizens | 0.08 | 0.14 | 0.95 | <0.001 | <0.001 | 0.58 |
| Mandatory | Incorporation of citizens’ contributions | 0.91 | 0.07 | 0.08 | <0.001 | 0.91 | 0.09 |
| **FDR for theme ‘’Responsibilities’’; typically no more than 1.7 false positives in abovementioned list of 35 rejections (Transformed P-value < 0.05, as FDR rate is set on 5%).** | | | | | | | |
