# Supplementary material for: Preferences for public engagement in decision-making regarding four COVID-19 non-pharmaceutical interventions in the Netherlands: A survey study
Source: PLoS One. 2023 Oct 5;18(10):e0292119. doi: 10.1371/journal.pone.0292119 (PMC10553365; doi:10.1371/journal.pone.0292119)
Supplement: S5 File — (DOCX) [file pone.0292119.s005.docx]

## Supplementary file 5 – Detailed results on suitable mode of engagement (Theme 2)

| S5. display of the percentages of respondents that ranked the five modes of engagement from least suitable (1) to most suitable (5). per rank, The most chosen mode of engagement is highlighted. | | | | | | | | | | | | | | | | | | | | |
| --- | --- | --- | --- | --- | --- | --- | --- | --- | --- | --- | --- | --- | --- | --- | --- | --- | --- | --- | --- | --- |
|  | Nightly curfew | | | | | Closure of schools & daycares | | | | | Covid entry pass | | | | | 1.5m social distancing | | | | |
|  | Inform | Consult | Advice | Collaborate | Empower | Inform | Consult | Advice | Collaborate | Empower | Inform | Consult | Advice | Collaborate | Empower | Inform | Consult | Advice | Collaborate | Empower |
| Rank 1 (least suitable) | 14% | 6% | 4% | 4% | 72% | 15% | 7% | 3% | 4% | 71% | 15% | 6% | 3% | 5% | 70% | 15% | 6% | 3% | 3% | 73% |
| Rank 2 | 12% | 18% | 20% | 44% | 5% | 12% | 18% | 22% | 43% | 5% | 11% | 19% | 20% | 45% | 5% | 11% | 19% | 19% | 46% | 4% |
| Rank 3 | 11% | 18% | 47% | 20% | 3% | 10% | 17% | 49% | 20% | 4% | 13% | 18% | 47% | 20% | 3% | 10% | 18% | 50% | 18% | 4% |
| Rank 4 | 16% | 41% | 19% | 20% | 4% | 13% | 44% | 19% | 21% | 3% | 15% | 42% | 21% | 19% | 4% | 14% | 44% | 19% | 20% | 3% |
| Rank 5 (most suitable) | 46% | 16% | 10% | 11% | 16% | 49% | 14% | 8% | 12% | 17% | 46% | 15% | 10% | 11% | 17% | 50% | 14% | 8% | 12% | 16% |
