# Supplementary material for: Preferences for public engagement in decision-making regarding four COVID-19 non-pharmaceutical interventions in the Netherlands: A survey study
Source: PLoS One. 2023 Oct 5;18(10):e0292119. doi: 10.1371/journal.pone.0292119 (PMC10553365; doi:10.1371/journal.pone.0292119)
Supplement: S6 File — (DOCX) [file pone.0292119.s006.docx]

## Supplementary file 6 – Results of Multinominal linear regression analysis

| S5. Detailed multinominal linear regression analysis with desire for engagement and demographic variables. significant results are highlighted in grey. | | | | | | | | | | | | | | | | | | | | |
| --- | --- | --- | --- | --- | --- | --- | --- | --- | --- | --- | --- | --- | --- | --- | --- | --- | --- | --- | --- | --- |
|  | Nightly curfew | | | | | Closure of schools & daycares | | | | | Covid entry pass | | | | | 1.5m social distancing | | | | |
|  | B | Sig. | Exp(B) | Lower bound | Upper bound | B | Sig. | Exp(B) | Lower bound | Upper bound | B | Sig. | Exp(B) | Lower bound | Upper bound | B | Sig. | Exp(B) | Lower bound | Upper bound |
| Gender |  | | | | | | | | | | | | | | | | | | | |
| Women | 0.486 | 0.000 | 1.625 | 1.341 | 1.969 | 0.138 | 0.213 | 1.148 | 0.924 | 1.428 | 0.404 | 0.000 | 1.497 | 1.236 | 1.814 | 0.397 | 0.000 | 1.487 | 1.211 | 1.824 |
| Age category |  | | | | | | | | | | | | | | | | | | | |
| 18-24 | -1.367 | 0.000 | 0.255 | 0.163 | 0.399 | -1.228 | 0.000 | .293 | 0.178 | 0.482 | -1.168 | 0.000 | 0.311 | 0.199 | 0.485 | -0,445 | 0.079 | 0.641 | 0.390 | 1.053 |
| 25-34 | -1.126 | 0.000 | 0.324 | 0.232 | 0.452 | -0,736 | 0.000 | 0.479 | 0.327 | 0.702 | -0,879 | 0.000 | 0.415 | 0.300 | 0.574 | -0,249 | 0.182 | 0.780 | 0,541 | 1,124 |
| 35-49 | -0,653 | 0.000 | 0.521 | 0.381 | 0.711 | -1,131 | 0.000 | 0.323 | 0.228 | 0.456 | -0,519 | 0.001 | 0.595 | 0.436 | 0.812 | -0,353 | 0.041 | 0.702 | 0.5 | 0.986 |
| 50-64 | -0,453 | 0.001 | 0.636 | 0.486 | 0.832 | -0,237 | 0.144 | 0.789 | 0.574 | 1.084 | -0,358 | 0.007 | 0.699 | 0.538 | 0.908 | -0,373 | 0.009 | 0.689 | 0.521 | 0.911 |
| Education level |  | | | | | | | | | | | | | | | | | | | |
| Low | -0,203 | 0.156 | 0.816 | 0.616 | 1.081 | 0.289 | 0.087 | 1.335 | 0.959 | 1.858 | -0,165 | 0.241 | 0.848 | 0.643 | 1.118 | -0,074 | 0.622 | 0.928 | 0.691 | 1.248 |
| Middle | -0,179 | 0.103 | 0.836 | 0.674 | 1.037 | -0,014 | 0.911 | 0.986 | 0.776 | 1.254 | -0,081 | 0.463 | 0.922 | 0.742 | 1.145 | -0,056 | 0.637 | 0.945 | 0.748 | 1.194 |
| Place of residency |  | | | | | | | | | | | | | | | | | | | |
| West | 0.204 | 0.089 | 1.226 | 0.969 | 1.551 | 0.237 | 0.084 | 1.268 | 0.969 | 1.659 | 0.223 | 0.062 | 1.250 | 0.988 | 1.581 | 0,06 | 0.641 | 1.062 | 0.825 | 1.368 |
| North | 0.249 | 0.165 | 1.283 | 0.903 | 1.824 | 0.246 | 0.227 | 1.279 | 0.858 | 1.905 | 0.217 | 0.230 | 1.242 | 0.872 | 1.768 | -0,209 | 0.260 | 0.811 | 0.564 | 1.168 |
| East | 0.249 | 0.085 | 1.283 | 0.966 | 1.704 | 0.036 | 0.822 | 1.036 | 0.758 | 1.417 | 0.461 | 0.002 | 1.586 | 1.187 | 2.120 | 0.168 | 0.284 | 1.183 | 0.870 | 1.608 |
| Migration Background |  | | | | | | | | | | | | | | | | | | | |
| Native | 0.077 | 0.671 | 1.080 | 0.756 | 1.545 | 0.465 | 0.027 | 1.591 | 1.055 | 2.399 | -0,092 | 0.634 | 0.912 | 0.624 | 1.334 | 0.386 | 0.054 | 1.471 | 0.993 | 2.180 |
| Non-Western Migrant | -0,707 | 0.026 | 0.493 | 0.265 | 0.917 | -0,333 | 0.357 | 0.716 | 0.353 | 1.456 | -0,732 | 0.027 | 0.481 | 0.251 | 0.920 | -0,219 | 0.519 | 0.803 | 0.412 | 1.564 |
