# Supplementary material for: Preferences for public engagement in decision-making regarding four COVID-19 non-pharmaceutical interventions in the Netherlands: A survey study
Source: PLoS One. 2023 Oct 5;18(10):e0292119. doi: 10.1371/journal.pone.0292119 (PMC10553365; doi:10.1371/journal.pone.0292119)
Supplement: Attachment — Submitted filename: Review 1.docx [file pone.0292119.s007.docx]

- A very important topic
- Reasonably excellent sample size of respondents
- More elaborations are needed on the (engagement in decision-making) types. Usually many government did not declare the (specifics) of who is most affected by COVID (i.e., age composition, more elderly’s, etc.).
- I the (Introduction) part, more details (and references) should be provided from (other countries) on the issue of (engaging the public). The practice of more countries should be included. Moreover, some more elaborations are needed on the (type of engagement) and how (candid) the interactions are.
- In the (study population) part, a relatively (short) time interval (period 27 October and 9 November 2021- driven by the Delta 114 variant) was mentioned. Any specific elaborations on why this time. Would the timing effect the results?
- In the (statistical analysis) part, it would be nice if you could provide the (Cronbach reliably) of each dimension, when possible. This could provide more (reliability) to the instrument used.
- In the (Results) section, more explanations of Table 2 is recommended.
- The (discussion) part needs to be elaborated upon more. It should cover (all) results obtained from the study. More comparison is also needed with other similar research with respect to results.
- In the (limitation) part, would you have recommended different analysis methods in future studies?
